# LACTATE CAN BE A MARKER OF METABOLIC SYNDROME IN SEVERE OBESITY?

**DOI:** 10.1590/0102-672020210001e1579

**Published:** 2021-06-11

**Authors:** Roberto DE-CLEVA, Lilian CARDIA, Alexandre VIEIRA-GADDUCCI, Julia Maria GREVE, Marco Aurelio SANTO

**Affiliations:** 1 Department of Gastroenterology, Faculty of Medicine, University of São Paulo, São Paulo, SP, Brazil; 2 Department of Orthopedics and Traumatology, Faculty of Medicine, University of São Paulo, São Paulo, SP, Brazil

**Keywords:** Obesity, Bariatric surgery, Lactic acid, Metabolic syndrome, Body composition, Obesidade, Cirurgia bariátrica, Ácido láctico, Síndrome metabólica, Composição corporal

## Abstract

**Background:**

In the last decades, numerous studies have confirmed the importance of lactate - by-product to the nutrient signal of the intracellular redox state - to regulatory functions in energy metabolism.

**Aim:**

To evaluate changes in blood lactate in patients with severe obesity and its correlation with body composition and metabolic profile.

**Methods:**

Twenty-four people with severe obesity (BMI=40 kg/m^2^) were evaluated in a prospective case-control study before and six months after Roux-in-Y gastric bypass. The blood lactate, total cholesterol, and fractions, C-reactive protein and HOMA-IR were analyzed after 12 h fasting. Body mass composition was evaluated by bioelectrical impedance and respiratory quotient was measured by indirect calorimetry.

**Results:**

The initial lactate level was 2.5±1.1 mmol/l and returned to normal level (1.9±3.6 mmol/l, p=0.0018) after surgery. This reduction was positively correlated with a decrease in BMI (p=0.0001), % free fat mass (p=0,001), % fat mass (p=0.001) and HOMA-IR (p=0.01). There was normalization of lactatemia in 70% of patients. There was no correlation between lactatemia and C-reactive protein.

**Conclusions:**

There was a significant improvement of metabolic parameters, normalization of blood lactate, fat mass loss, although these individuals remained with a high BMI.

## RESUMO

**Racional:**

Nas últimas décadas, inúmeros estudos confirmam a importância do lactato - subproduto ao sinal de nutrientes do estado redox intracelular - às funções reguladoras do metabolismo energético.

**Objetivo:**

Avaliar alterações no lactato sanguíneo em pacientes com obesidade grave e sua correlação com a composição corporal e o perfil metabólico.

**Métodos:**

Vinte e quatro pacientes com obesidade grave (IMC=40 kg/m^2^) foram avaliadas em um estudo prospectivo antes e após seis meses do bypass gástrico em Y-de-Roux. O lactato sanguíneo, colesterol total e frações, proteína C-reativa e HOMA-IR foram analisados após 12h de jejum. A composição corporal foi avaliada por bioimpedância elétrica e o quociente respiratório por calorimetria indireta.

**Resultados:**

O nível inicial de lactato foi de 2,5±1,1 mmol/l e retornou ao nível normal após a operação (1,9±3,6 mmol/l, p=0,0018). Essa redução foi correlacionada positivamente com a diminuição do IMC (p=0,0001), % de massa livre de gordura (p=0,001) e % de massa de gordura (p=0,001) e HOMA-IR (p=0,01). Houve normalização da lactatemia em 70% dos pacientes. Não houve correlação entre lactatemia e proteína C-reativa.

**Conclusões:**

Houve melhora significativa dos parâmetros metabólicos e normalização da lactatemia, perda de massa gorda, embora esses indivíduos permanecessem com IMC elevado.

## INTRODUCTION

The increasing prevalence of obesity and metabolic syndrome (MetS) has become a concern, mainly due to risk of progression to type 2 diabetes and cardiovascular diseases^15^ ^,^ ^2^ ^,^ ^29^.

Since the first descriptions, MetS and increased resistance to insulin are interrelated^11^ ^,^ ^26^, although this variable is difficult to assess in the clinical context. As consequence, clinical characteristics of insulin resistance, such as abdominal obesity, dyslipidemia, hypertension, hyperinsulinemia and glucose intolerance are used to compose its concept. Other features of MetS are a low-grade systemic inflammatory state, a prothrombotic state, and metabolic inflexibility, which is the loss of the body’s ability to adapt the consumption of substrates to its availability^3^. If we consider MetS and severe obesity from the point of view of metabolic flexibility, a compelling perspective is opened to study the role of lactate in this context. In the last decades, numerous studies have confirmed the importance of lactate - by-product to the nutrient signal of the intracellular redox state - with important regulatory functions in energy metabolism ^23,10,30,22.^

Despite the extensive literature on the interrelationships between adipose tissue and lactate, there are virtually no prospective clinical studies evaluating the prevalence of increased basal lactate in patients with severe obesity individuals as well as the impact of weight loss in this context.

The aim of this study was to determine changes in blood lactate level at rest in subjects with severe obesity before and one-year after bariatric surgery as well as correlations between blood lactate concentration, body composition (lean and body fat mass) and improvement in metabolic profile.

## METHODS

A prospective case control study with 24 patients with severe obesity (BMI=40 kg/m^2^) before and six months after bariatric surgery at Metabolic and Bariatric Unit Hospital das Clínicas, University of São Paulo Medical School, São Paulo, SP, Brazil. The current study was performed according to the ethical recommendations of the Declaration of Helsinki, and it was approved by the Hospital das Clínicas Ethical Committee (n^o^. 01038912600000068)

 Inclusion criteria were BMI>40 kg/m^2^, age between 18 and 60 years, absence of type 2 diabetes (American Diabetes Association - 2012), chronic lung disease and limiting cardiopathy. 

### Blood lactate concentration

For its determination, blood samples were taken from the finger in the morning after 12 h fasting using Accu-Chek^®^ (Diagnostics GmbH Roche, Germany). Lactate levels were measured by placing a drop of blood on the test strip BM-Lactate (Roche Diagnostics GmbH, Germany) and introducing it in Accutrend^®^ Lactate (Roche Diagnostics GmbH, Germany).

### Biochemical data

Samples for laboratory tests were also collected in the morning after a 12 h fasting. Insulin resistance measurements were obtained using the HOMA-IR model. All biochemical measurements were performed from plasma samples obtained by centrifugation of whole blood (3,000 rpm, 20 min, temp =4.0° C). Insulin levels and C-peptide were determined by MO electrochemistry. The levels of total cholesterol and fractions (LDL-c, VLDL-c and HDL-c) were determined by CHOD/PAP ID-MS colorimetric enzymatic method. Ultrasensitive C-reactive protein (CRP) was determined by immunoturbimetric assay.

### Anthropometric measurements, body mass composition and respiratory quotien

Body composition was evaluated by bioelectrical impedance analysis (BIA) using InBody 230^®^ (GE Healthcare, USA) and anthropometry. Height and body weight were measured with participants to the nearest 0.5 cm and 0.1 kg, respectively. Body mass index (BMI) was calculated using the formula: weight (kg)/height (m^2^). Respiratory quotient (RQ) was calculated from the ratio between total CO_2_ production and O_2_ consumed and was measured by indirect calorimetry using the Med Graphics System, Ultima CPX (Medgraphics, St. Paul, MN, USA).

### Surgical procedure

The procedure performed was gastric bypass in Roux-en-Y, with biliopancreatic loop 60-75 cm and food loop of 100-120 cm, with 30 ml volume pouch.

### Statistical analysis

The results of descriptive analysis were expressed as mean ± standard deviation for quantitative variables and percentages for qualitative variables and p<0.05 were considered as significant. Statistical analysis was performed using R program version 2010.

## RESULTS

The clinical characteristics and laboratory data from patients before and six months after bariatric surgery are shown in Table 1.

**TABLE 1:** Anthropometrics, body composition and laboratory data of patients with severe obesity

	Normal values	Pre-op	**Pos-op**	p
BMI (kg/m^2^)	18.5 - 25.0	48.9±5.1	35.6±4.9	<0.0001
FFM (%)	75 - 90	51.2±4.0	66.3±8.0	<0,001
FM (%)	10 - 25	48.8±4.7	35.7±4.9	<0.001
Lactate	<2 mmol/l	2.5±1.1	1.9±0.7	<0.001
Glycemia	70 - 100 mg/dl	101.0±28.7	90.0±12.0	<0.01
Insulin	2.6-24.9 mcU/ml	31.9±27.6	8.9±4.6	<0.001
HOMA-IR	<3.4	8.67±9.17	2.0±1.4	<0.01
C-peptide	1.1- 4.4 ng/ml	4.2±2.0	2.3±0.8	<0.001
C-reactive protein	<5 mg/l	14.4±8.7	2.0±2.1	<0.001
Triglycerides	<150 mg/dl	136.0±105.7	81.7±29.2	<0.01
Cholesterol	<200 mg/dl	171.8±32.3	157.6±27.5	<0.01
HDL-c	>60 mg/dl	45.0±9.6	54.9±13.0	<0.01
LDL-c	<100 mg/dl	105.9±37.1	92.1±33.4	<0.10
TG/HDL-c	Male <2.5	3.4±3.3	1.6±0.8	<0.01
	Female <3.5			

BMI=body mass index; FFM=free-fat mass; FM=fat mass

Female were in 83% of the individuals. The mean age of the participants was 38.5±9.6 years. The initial blood lactate level at rest was 2.5±1.1 mmol/l. They also had insulin resistance based on HOMA-IR values. Resting lactate values returned to the normal level (1.9±3.6 mmol/l, p=0.0018) after surgery and this reduction was positively correlated with a decrease in BMI (p<0.0001), percentage of free fat mass (p<0.001), total fat mass (p<0.001) and HOMA-IR index (p<0.01).

## DISCUSSION

The mechanisms responsible for metabolic changes associated with MetS and obesity are complex and still poorly understood. Disorders of fat metabolism decreased arterial flow to white adipose tissue (WAT) and the installation of a low-grade inflammatory state secondary to hypoxia appears to be the triggering events of the metabolic changes that accompany MetS, such as increased resistance to insulin action, hyperglycemia and hyperlactatemia^28^. Initially, it was attempted to demonstrate that tissue hypoxia at the level of WAT would be the cause of increased local production of lactate, similarly to what would happen in the musculature under anaerobic conditions (oxygen deficit theory)^13^. 

Nevertheless, lactate is not simply the end result of glucose oxidation under anaerobic conditions. In fact, some situations of high energy demand, in addition to replacing blood glucose and hepatic glycogen, lactate also participates in the regulation of peripheral glucose metabolism^4^. 

It has even been suggested that it acts as a hormone, modifying the expression of several enzymes and transporters related to cellular bioenergetics, inducing the release of insulin from the beta cells and increasing the pancreatic response to insulin secretagogues^10^ or inhibiting lipolysis^17^. The great responsible for this new point of view was the discovery of the monocarboxylic acid carriers (MCT) coupled to the transport of the hydrogen ion^27^. 

The expression of these transporters in the various tissues varies in response to multiple factors such as oxygen availability at the cellular level, changes in exercise routine, muscle regeneration processes, changes in dietary pattern, prolonged fasting and obesity that ultimately modifies the lactate kinetics according to the demands of energy metabolism ^33^. 

Resting lactatemia (up to 2 mmol/l) is the result of a balance between constant production and clearance (approximately 1 mmol/kg/h of lactate) produced in various organs and tissues^1^. The WAT contributes significantly to this kinetics, with variations related to ethnicity, anatomical region studied (visceral vs. subcutaneous adipose tissue, e.g.), level of physical activity, nutritional status and insulin action ^7^.

In this series with severe obesity had an increase in lactate at rest and fasting (2.5±1.1 mmol/l), with point values that reached 4.6 mmol/l, similar to values found in individuals submitted to physical exercise with intensity close to the anaerobic threshold ^24^.

In healthy non-obese subjects, approximately 30% of the glucose absorbed by subcutaneous adipose tissue during fasting is converted to lactate by non-oxidative metabolism. After carbohydrate intake, lactatemia rises following the elevation of the blood level of insulin, with up to 23.5% of the circulating glucose removed WAT in this period being converted into lactate^6^. As obesity progresses (increased fat mass), there are important changes in glucose to lactate metabolism, as well as glucose intolerance and dyslipidemia ^19^. 

In the opposite direction was noticed a significant normalization of lactatemia in 70% of the patients after the reduction of the fat mass (66.4±10.7 kg to 38.3±11.3 kg). It is interesting to note that even after a significant loss of fat mass, the mean BMI was 35.6±4.9 kg/m^2^, perhaps the reason why lactatemia did not normalize in all patients.

Resting hyperlactatemia is an indicator of low oxidative capacity and is associated with an increased risk of cardiac failure, atherosclerosis, arterial hypertension, type 2 diabetes and insulin resistance, probably as a consequence of decreased capacity of oxidative phosphorylation due to mitochondrial dysfunction ^21^.

The increase in HIF-1alpha in response to hypoxemia induces several modifications in energy metabolism. Initially it stimulates the expression of glucose transporter type 4 (GLUT4) and up regulates enzymes from the glycolytic pathway and activates lactate dehydrogenase, which recycles NAD+ and allows the continuity of glycolysis activity. In addition, it induces pyruvate dehydrogenase kinase 1, resulting in blockade in the conversion of pyruvate-acetyl-CoA, determining decrease in Krebs activity and greater availability of pyruvate for conversion to lactate^18^. 

There was a significant decrease (from 1.12±0.09 to 0.98±0.06) in RQ of the patients before and after the loss of fat mass, which could mean a trend towards a less glycolytic metabolism with a recovery of the oxidative capacity. 

Experimental research in fat cell culture, was suggested that the massive production of lactate is a cellular defense process, used to eliminate excess glucose, exporting the carbon from glucose to the liver. In addition to circumventing the problem of excess glucose availability, this mechanism helps control hyperglycemia in the short term ^20^. 

The glucose and lactate metabolism alterations associated with obesity are not restricted to WAT. The basal muscle release of lactate is significantly higher in individuals with obesity when compared to non-obese controls in skeletal muscle preparations^1^. The skeletal muscle of individuals with obesity, when compared to that of non-obese individuals, presents several structural and functional changes such as sarcopenia^12^, muscle remodeling with an increase in the density of glycolytic muscle fibers^14^ and a tendency in the increase of the expression of the monocarboxylate transporters 4 (MTC) responsible for the extrusion of large amounts of lactate, probably an adaptive mechanism to their physical condition. Caloric restriction and weight loss induce a slight decrease in the level of expression of the MTC4, towards control values ^11^. 

In addition, the quantitative proteomic analysis of the skeletal muscle of obese individuals shows an increase in the content of glycolytic enzymes and down regulation of mitochondrial proteins, suggesting a decrease in oxidative metabolism^10^. 

It is well known that in other conditions associated with an increase in lactatemia (as in hyperlactatemia of stress), it represents a refined mechanism of adaptive response to oscillations in energy demand than a simple metabolic out of control produced by desoxya^14^. With obesity, in addition to the absolute increase in fat mass, there is an increase in basal lactate production per gram of fat tissue^8^, although its release in response to glucose infusion is depressed^5^ **.**

There is an inverse relationship between insulin sensitivity index and baseline lactate level, and it is still controversial whether obesity-related hyperlactatemia is a consequence of insulin resistance, exclusively due to obesity itself or associated with other factors^16^. In our patients, was found a significant fall in insulinemia and normalization of HOMA-IR after surgery, although was not percieved any association between the findings.

The low-grade inflammatory state that is established in obese individuals is probably one of the main factors responsible for the extra metabolic changes in the WAT^32^. As a consequence of the mismatch between the effective capillary blood flow and the fat mass, a desoxy environment with loss of homeostasis control infiltration of macrophages and development of a low-grade inflammatory state at the WAT level. There is also local release of free radicals that together induce local and systemic metabolic alterations such as dysregulation in the expression of cartilage matrix deficiency in various organs, remodeling of skeletal muscular tissue and increased resistance to the action of insulin MTC1 dysfunction is a key factor associated to the late occurrence of insulin resistance^31^. 

As observed in other studies, our patients presented normalization of C-reactive protein after weight loss^25^ (Table 1), suggesting a decrease in inflammatory activity. Although we did not find a correlation between normalization of lactatemia and C-reactive protein, it is possible that the improvement in the inflammatory state, reflected by the decrease in it is correlated with the improvement in lactatemia^9^.

Further studies are needed to determine if these phenomena are interconnected, whether blood lactate levels could be considered a marker of the metabolic disorders associated with obesity and what their real significance in metabolism in individuals with severe obesity.

## CONCLUSION

The rest hyperlactatemia observed in patients with severe obesity is accompanied by hyperinsulinemia, dyslipidemia and low grade chronic inflammatory state. Was found a significant improvement in these parameters, with up to 70% normalization with loss of fat mass after Roux-in-Y gastric bypass, although these individuals remained with a high BMI.
